# A comparison of standard and high dose adenosine protocols in routine vasodilator stress cardiovascular magnetic resonance: dosage affects hyperaemic myocardial blood flow in patients with severe left ventricular systolic impairment

**DOI:** 10.1186/s12968-021-00714-7

**Published:** 2021-03-18

**Authors:** Louise A. E. Brown, Christopher E. D. Saunderson, Arka Das, Thomas Craven, Eylem Levelt, Kristopher D. Knott, Erica Dall’Armellina, Hui Xue, James C. Moon, John P. Greenwood, Peter Kellman, Peter P. Swoboda, Sven Plein

**Affiliations:** 1 Multidisciplinary Cardiovascular Research Centre (MCRC) & Biomedical Imaging Science Department, Leeds Institute of Cardiovascular and Metabolic Medicine, University of Leeds, Clarendon Way, Leeds, LS2 9JT UK; 2 The Cardiovascular Magnetic Resonance Imaging Unit and The Inherited Cardiovascular Diseases Unit, Barts Heart Centre, St Bartholomew’s Hospital, West Smithfield, London, UK; 3 National Heart, Lung, and Blood Institute, National Institutes of Health, DHHS, Bethesda, MD USA

**Keywords:** Perfusion, Myocardial blood flow, Adenosine stress, Heart failure

## Abstract

**Background:**

Adenosine stress perfusion cardiovascular magnetic resonance (CMR) is commonly used in the assessment of patients with suspected ischaemia. Accepted protocols recommend administration of adenosine at a dose of 140 µg/kg/min increased up to 210 µg/kg/min if required. Conventionally, adequate stress has been assessed using change in heart rate, however, recent studies have suggested that these peripheral measurements may not reflect hyperaemia and can be blunted, in particular, in patients with heart failure. This study looked to compare stress myocardial blood flow (MBF) and haemodynamic response with different dosing regimens of adenosine during stress perfusion CMR in patients and healthy controls.

**Methods:**

20 healthy adult subjects were recruited as controls to compare 3 adenosine perfusion protocols: standard dose (140 µg/kg/min for 4 min), high dose (210 µg/kg/min for 4 min) and long dose (140 µg/kg/min for 8 min). 60 patients with either known or suspected coronary artery disease (CAD) or with heart failure and different degrees of left ventricular (LV) dysfunction underwent adenosine stress with standard and high dose adenosine within the same scan. All studies were carried out on a 3 T CMR scanner. Quantitative global myocardial perfusion and haemodynamic response were compared between doses.

**Results:**

In healthy controls, no significant difference was seen in stress MBF between the 3 protocols. In patients with known or suspected CAD, and those with heart failure and mild systolic impairment (LV ejection fraction (LVEF) ≥ 40%) no significant difference was seen in stress MBF between standard and high dose adenosine. In those with LVEF < 40%, there was a significantly higher stress MBF following high dose adenosine compared to standard dose (1.33 ± 0.46 vs 1.10 ± 0.47 ml/g/min, p = 0.004). Non-responders to standard dose adenosine (defined by an increase in heart rate (HR) < 10 bpm) had a significantly higher stress HR following high dose (75 ± 12 vs 70 ± 14 bpm, p = 0.034), but showed no significant difference in stress MBF.

**Conclusions:**

Increasing adenosine dose from 140 to 210 µg/kg/min leads to increased stress MBF in patients with significantly impaired LV systolic function. Adenosine dose in clinical perfusion assessment may need to be increased in these patients.

## Introduction

Stress perfusion cardiovascular magnetic resonance (CMR) is an accurate, non-invasive technique for the detection of myocardial ischaemia [1, 2]. The method is widely used in the assessment of patients with suspected or known coronary artery disease (CAD); either to detect ischaemia, or in the context of cardiac dysfunction to detect an underlying ischaemic cause.

Intravenous adenosine has been shown to induce near maximal hyperaemia [3] and is used for assessment of ischaemia using both invasive measurements such as fractional flow reserve (FFR) and non-invasive techniques including CMR and positron emission tomography (PET). For stress perfusion CMR, adenosine is the most commonly used pharmacological stress agent [4]. Accepted protocols recommend administration of adenosine at a dose of 140 µg/kg/min with an increase up to 210 µg/kg/min if required to achieve adequate stress [5]. The duration of adenosine infusion is standardised and usually given for at least 3 min prior to data acquisition, but it is not known if a longer duration or higher dose may produce a better response.

Conventionally, adequate stress is defined by a heart rate (HR) rise of ≥ 10 bpm or a systolic blood pressure (SBP) fall of > 10 mmHg [5], based on the assumption that coronary vasodilatation leads to systemic vasodilation and reflex tachycardia. Recent studies have suggested however, that these peripheral measurements may not be a true reflection of myocardial hyperaemia and should not be used to assess adenosine response [6, 7]. In addition, certain patient groups including those with heart failure and diabetes mellitus have a blunted haemodynamic response to intravenous adenosine [8–10] and it is unclear to what extent this reduced response is reflected in coronary vasodilation.

Recently developed techniques of inline myocardial perfusion mapping with CMR provide accurate, reproducible assessment of rest and vasodilator stress myocardial blood flow (MBF) following adenosine administration [11–13]. This study looked to compare stress MBF with different dosing regimens of adenosine during stress perfusion CMR in patients with suspected CAD, heart failure and healthy controls.

## Methods

### Study population

Twenty healthy subjects were recruited as controls to compare 3 adenosine perfusion protocols. Exclusion criteria were any known cardiovascular disease, hypertension, hyperlipidaemia, diabetes mellitus, smoking, body mass index (BMI) > 30 and any contraindication to CMR, adenosine or gadolinium-based contrast agents.

Sixty patients with symptoms of angina or heart failure were recruited prospectively from CMR or coronary angiography waiting lists, for comparison of 2 adenosine protocols determined from the results in healthy subjects. Patients were divided into three groups for analysis consisting of; Group 1—Patients with coronary artery disease and left ventricular (LV) ejection fraction (LVEF) ≥ 40%. Group 2—Mild to moderate heart failure, LVEF ≥ 40% and no evidence of coronary disease, and Group 3—Moderate to severe heart failure, LVEF < 40% and no evidence of CAD. Exclusion criteria for patients were the presence of any contraindication to CMR, adenosine or gadolinium-based contrast agents. CAD was defined by either ischaemic (subendocardial) late gadolinium enhancement (LGE) on CMR, at least moderate stenosis on coronary angiography, previous percutaneous coronary intervention or previous coronary arterial bypass grafting.

### Study protocol

All CMR studies were undertaken on a 3 T CMR system (Magnetom Prisma, Siemens Healthineers, Erlangen, Germany). Participants were advised to avoid caffeine for 24 h before the study. The protocol consisted of cine imaging, stress and rest perfusion, and LGE.

For perfusion imaging, adenosine was infused at a set dose for a preassigned time. Healthy subjects had three stress perfusion acquisitions; standard dose (140 µg/kg/min adenosine for 4 min), high dose (210 µg/kg/min for 4 min) and long dose (140 µg/kg/min for 8 min). Patients received standard dose and high dose for 4 min each. Doses were given in random order. A 10-min interval was kept between perfusion acquisitions, rest perfusion images were acquired 10 min after the final stress perfusion sequence in all participants.

Participants were monitored for symptoms throughout the scan. SBP and HR were recorded prior to starting adenosine infusion and before acquisition. For each perfusion acquisition, an intravenous bolus of 0.05 mmol/kg gadobutrol (Gadovist, Bayer Healthcare, Berlin, Germany) was administered at 5 ml/s followed by a 20 ml saline flush using an automated injection pump (Medrad MRXperion Injection System, Bayer Healthcare). Perfusion mapping was performed and implemented on the scanner using the Gadgetron streaming software image reconstruction framework as previously described [11, 13]. MBF maps were acquired as a short axis stack using a free-breathing, dual sequence, saturation recovery fast low angle shot (FLASH) protocol with motion correction. Three 8 mm slices were acquired, with slice spacing varied on a per patient basis to cover the left ventricle.

### Analysis

Ischaemic segments were identified on visual assessment of perfusion images. Splenic switch off was assessed by comparing enhancement of splenic tissue at stress and at rest according to previously published methods [14, 15].

Perfusion maps were analysed using cvi42 software (Circle Cardiovascular Imaging, Calgary, Alberta, Canada). Endocardial and epicardial borders were drawn excluding papillary muscles, right ventricular insertion points marked, and a 16-segment American Heart Association model [16] used for further segmentation. In order to minimise partial volume effect, a 10% offset was applied to endocardial and epicardial borders [12]. MBF was recorded for each of the 16 segments. Where the LV outflow tract was included, or segments were too thin to contour, these segments were excluded from further analysis. Segments with ischaemic (subendocardial) LGE were also excluded from analysis. MBF values for all remaining segments were averaged to provide a value for global MBF.

### Subgroup analyses

Subgroup analysis was carried out comparing patients with a HR change of < 10 bpm (non-responders) compared to those with a rise of ≥ 10 bpm (responders). Within Group 1, segments with ischaemia on visual assessment were compared with non-ischaemic segments.

### Statistical analysis

Analysis was performed using SPSS (version 23, Statistical Package for the Social Sciences, International Business Machines, Inc., Armonk, New York, USA). Normality of distribution was assessed using Shapiro–Wilk test. Different dosing regimens were analysed using paired t-tests or Wilcoxson Signed Rank test in patients and analysis of variance (ANOVA) with repeated measures and post-hoc Bonferroni correction, or the Kruskal Wallis H test in healthy controls. Categorical data was analysed using chi-square test or Fisher’s exact test when expected numbers were < 5. All statistical tests were two-tailed and p < 0.05 was considered significant.

Myocardial perfusion reserve (MPR) was calculated as stress MBF:rest MBF. Inadequate HR response was defined as < 10 bpm in keeping with Society for Cardiovascular Magnetic Resonance guidelines [5].

## Results

### Healthy controls

20 healthy control subjects were recruited (25 ± 2.7 years, LVEF 57 ± 3.3%). One subject withdrew after the first dose of adenosine. Haemodynamic data were available for all three dosing regimens for 19 healthy subjects. In one case artefact on perfusion maps at standard dose meant these were not included in analysis of MBF response. The final cohort therefore consisted of 18 subjects.

### Haemodynamic response

Haemodynamic data are shown in Table 1. For all doses there was a significant rise in HR at stress from rest (p < 0.001). In both standard and long dose protocols there was a significant increase in stress SBP from rest (p < 0.01). There was no significant change in SBP between rest and high dose adenosine. Only one participant had a SBP decrease of ≥ 10 mmHg at standard and high dose adenosine, and none had this degree of change with long dose.

**Table 1:** Haemodynamic response and MBF in healthy subjects

	Adenosine dosing			
	Standard dose: 140 µg/kg/min 4 min duration	High dose: 210 µg/kg/min 4 min duration	Long dose: 140 µg/kg/min 8 min duration	p
Rest HR (bpm)	71 ± 15	71 ± 14	71 ± 13	0.962
Rest SBP (mmHg)	117 ± 12	118 ± 14	117 ± 13	0.481
Stress HR (bpm)	102 ± 19^†^	108 ± 16^†^	106 ± 19^†^	0.017*
Stress SBP (mmHg)	121 ± 16^†^	121 ± 16	124 ± 17^‡^	0.195
Change in HR (bpm)	30 ± 12	37 ± 11	35 ± 13	0.045*
Change in SBP (mmHg)	5 ± 6	3 ± 9	7 ± 11	0.072
Stress MBF (ml/g/min)	2.50 ± 0.74	2.66 ± 0.59	2.59 ± 0.64	0.323
MPR	3.52 ± 0.93	3.82 ± 0.83	3.72 ± 1.00	0.191

A significant difference was seen in stress heart rate and change in stress HR

*HR* heart rate, *SBP* systolic blood pressure, *MBF* myocardial blood flow, *MPR* myocardial perfusion reserve

^*^Significant difference between adenosine doses, ^†^significant difference from rest value, p < 0.05 ^‡^significant difference from rest value, p < 0.01. Data given as mean ± standard deviation

There was a significant difference in stress HR and between standard and high dose adenosine, but no difference between standard and long dose. No significant difference was seen in SBP or change in SBP between doses.

### MBF

Global stress MBF was 2.50 ± 0.74 ml/g/min with standard dose adenosine, with values of 2.66 ± 0.59 ml/g/min after high dose and 2.59 ± 0.64 ml/g/min with long dose, overall there was no significant difference between the three groups (p = 0.32).

**Fig. 1:**
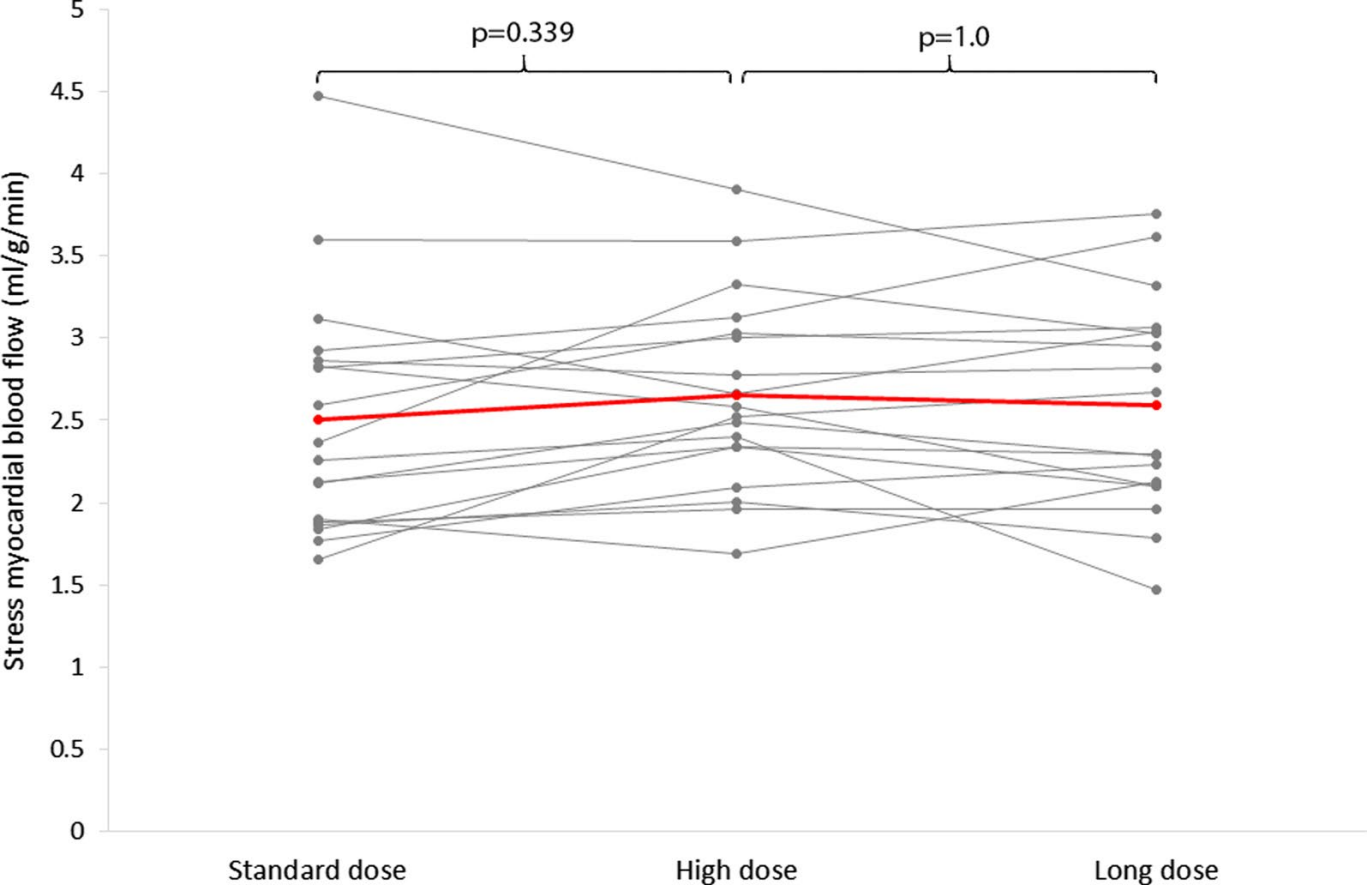
Results in healthy control group (n = 19). **a** Increase in heart rate (HR) between adenosine doses, **b** stress myocardial blood flow (MBF) between adenosine doses. In healthy subjects, no significant differences in MBF were seen between doses of adenosine despite significantly higher HR with high dose adenosine and long dose adenosine

Although there was no significant difference in MBF, the largest increases in HR were seen following the high dose regimen (Fig. 1), therefore this regime was chosen for comparison with standard dose in patients.

### Patients

Sixty patients took part in the study divided into 20 in Group 1 (CAD), 16 in Group 2 (mild to moderate heart failure, LVEF ≥ 40%) and 24 in Group 3 (moderate to severe heart failure, LVEF < 40%). No significant differences were seen between the groups in incidence of diabetes mellitus, beta-blocker usage, age or sex (Table 2).

**Table 2:** Characteristics of patient groups

	Group 1	Group 2	Group 3	p
n	20	16	24	
Sex—male	17 (85%)	8 (50%)	17 (71%)	0.074
Age	62.9 ± 8.7	63.5 ± 16.3	65.1 ± 12.7	0.344
Diabetes mellitus	4 (20%)	0	6 (25%)	0.102
Beta blocker usage	14 (70%)	12 (75%)	17 (71%)	0.940
LVEF	57.5 ± 7.9	48.1 ± 5.2	26.2 ± 7.0	< 0.001

Other than left ventricular ejection fraction (LVEF), no significant difference was seen between the groups in factors previously reported to affect adenosine response

Data given as mean ± standard deviation or n (%)

Group 1—Patients with coronary disease and left ventricular ejection fraction (LVEF) > 40%. Group 2—Mild to moderate heart failure, LVEF ≥ 40% and no evidence of coronary artery disease. Group 3—moderate to severe heart failure, LVEF < 40% and no evidence of coronary artery disease

### Haemodynamic response

Haemodynamic data are shown in Table 3. Mean stress HR increased significantly from mean rest HR in all groups and following both standard and high dose adenosine (p < 0.01). There was no significant change between stress and rest SBP in any group. In total, 36 (60%) patients had a HR rise ≥ 10 bpm with standard dose adenosine, and 42 (70%) with high dose. One (2%) patient had a SBP decrease of ≥ 10 mmHg with standard dose, and 2 (3%) with high dose. In each group, there was a similar proportion of non-responders.

**Table 3:** Haemodynamics and response to adenosine

	CAD n = 20			HF LVEF ≥ 40% n = 16			HF LVEF < 40% n = 24			Comparison between groups (p)	
	Standard dose	High dose	p	Standard dose	High dose	p	Standard dose	High dose	p	Standard dose	High dose
Rest HR (bpm)	62 ± 10	63 ± 9	0.534	65 ± 14	65 ± 13	1.000	75 ± 12	77 ± 14	0.547	0.002	0.001
Rest SBP (mmHg)	132 ± 17	131 ± 16	0.735	124 ± 16	124 ± 17	0.320	119 ± 16	119 ± 16	0.526	0.066	0.089
Stress HR (bpm)	75 ± 13^‡^	78 ± 13^‡^	0.027*	79 ± 20^‡^	80 ± 17^‡^	0.659	87 ± 16^‡^	90 ± 18^‡^	0.418	0.039	0.038
Stress SBP (mmHg)	124 ± 15	121 ± 12	0.219	124 ± 11	120 ± 13	0.005**	122 ± 18	124 ± 19	0.391	0.597	0.970
Change in HR (bpm)	12 ± 10	15 ± 8	0.112	14 ± 14	15 ± 9	0.637	12 ± 12	13 ± 19	0.751	0.875	0.889
Change in SBP	− 2 ± 10	− 6 ± 11	0.455	− 2 ± 13	− 2 ± 11	0.139	2 ± 8	3 ± 13	0.700	0.377	0.072
Non-responders (< 10 bpm) (%)	8 (40%)	4 (20%)	0.168	6 (38%)	3 (19%)	0.433	10 (42%)	11 (46%)	0.771	0.966	0.097
Failed splenic switch off (%)	2 (10%)	0	0.487	1 (6%)	0	1	2 (8%)	2 (8%)	1	1.0	0.334

Resting HR was significantly higher in the moderate-severe heart failure (HF) group, no difference was seen between groups in rate of non-responders or absolute measures of haemodynamic response to adenosine

Data given as mean ± standard deviation or n (%)

*HR* heart rate, *SBP* systolic blood pressure

^*^Significant difference between adenosine doses, p < 0.05, **significant difference between adenosine doses, p < 0.01, ^‡^significant difference from rest value, p < 0.01. Group 1—Patients with coronary artery disease and left ventricular ejection fraction (LVEF) > 40%. Group 2 – Mild to moderate heart failure, LVEF ≥ 40% and no evidence of coronary artery disease. Group 3—moderate to severe heart failure, LVEF < 40% and no evidence of coronary artery disease. Standard dose—140 µg/kg/min. High dose—210 µg/kg/min.

Within group 1, stress HR was significantly higher following high dose compared with standard dose adenosine (78 ± 13 vs 75 ± 13 bpm, p = 0.025), but no significant difference was seen in groups 2 or 3. Stress SBP was significantly lower following high dose adenosine compared to standard dose in group 2 (120 ± 13 vs 124 ± 11 mmHg, p = 0.005). There was no significant difference in stress SBP between doses in the other groups.

**Fig. 2:**
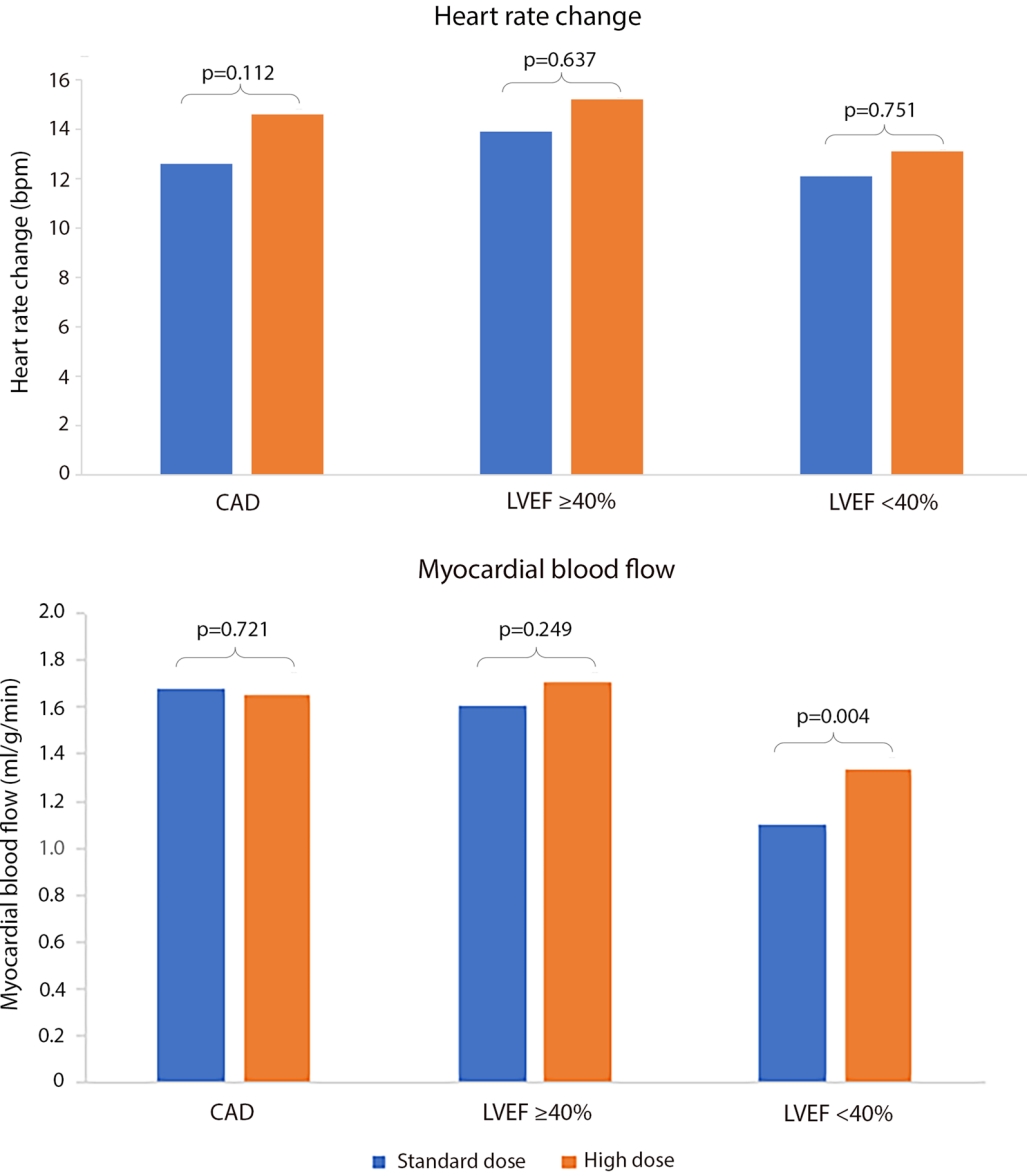
While high dose adenosine did not result in significantly larger heart rate changes in any of the three groups of patients, high dose adenosine did improve stress MBF in patients with left ventricular ejection fraction (LVEF) < 40%

No significant difference was seen in absolute HR rise between the groups (Fig. 2) and no significant correlation was seen between LVEF and HR rise across all 60 patients (r = 0.122, p = 0.353).

### MBF and MPR

**Fig. 3:**
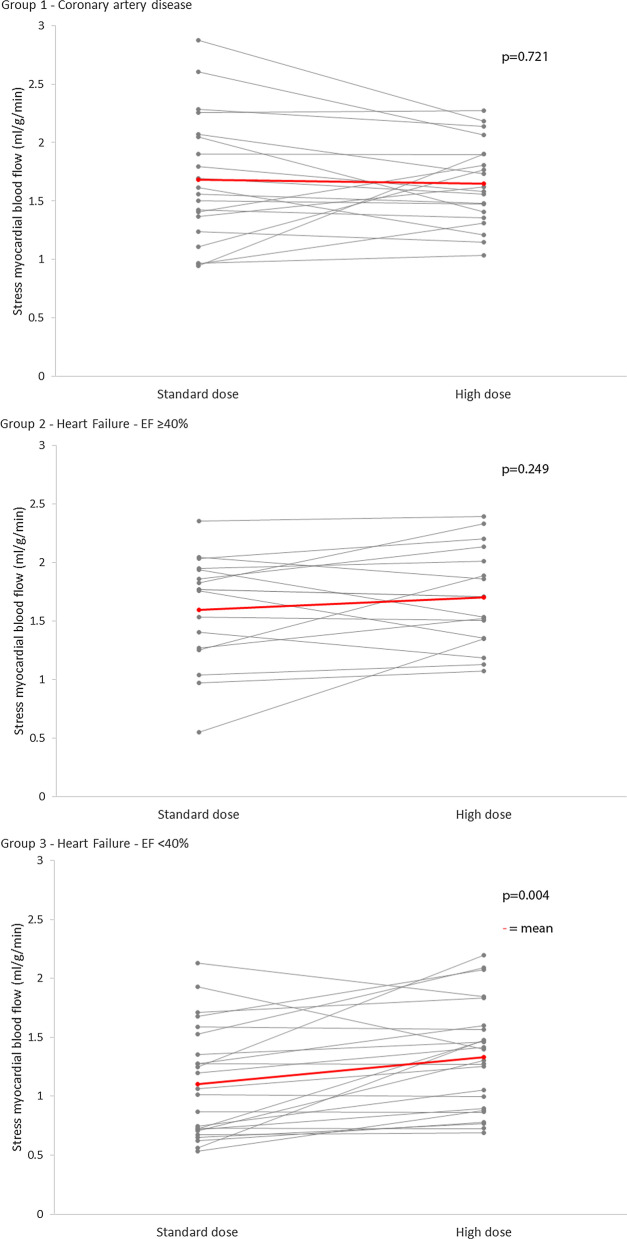
Difference in stress MBF between doses in three groups of patients. Within Group 3 (moderate-severe heart failure) stress MBF was significantly higher with high dose adenosine compared to standard dose

No difference was seen in rest MBF between the 3 groups (Group 1—0.73 ± 0.16 ml/g/min, Group 2—0.71 ± 0.23 ml/g/min, Group 3—0.62 ± 0.16 ml/g/min, p = 0.144). No difference was seen in MBF or MPR between different adenosine doses in either Group 1 or 2. Within Group 3, MBF was significantly higher following high dose than after standard dose adenosine (standard dose 1.10 ± 0.47 vs high dose 1.33 ± 0.46 µg/ml/min, p = 0.004) (Figs. 2, 3). MPR demonstrated the same pattern (standard dose 1.90 ± 0.88 vs high dose 2.26 ± 0.90, p = 0.004).

**Fig. 4:**
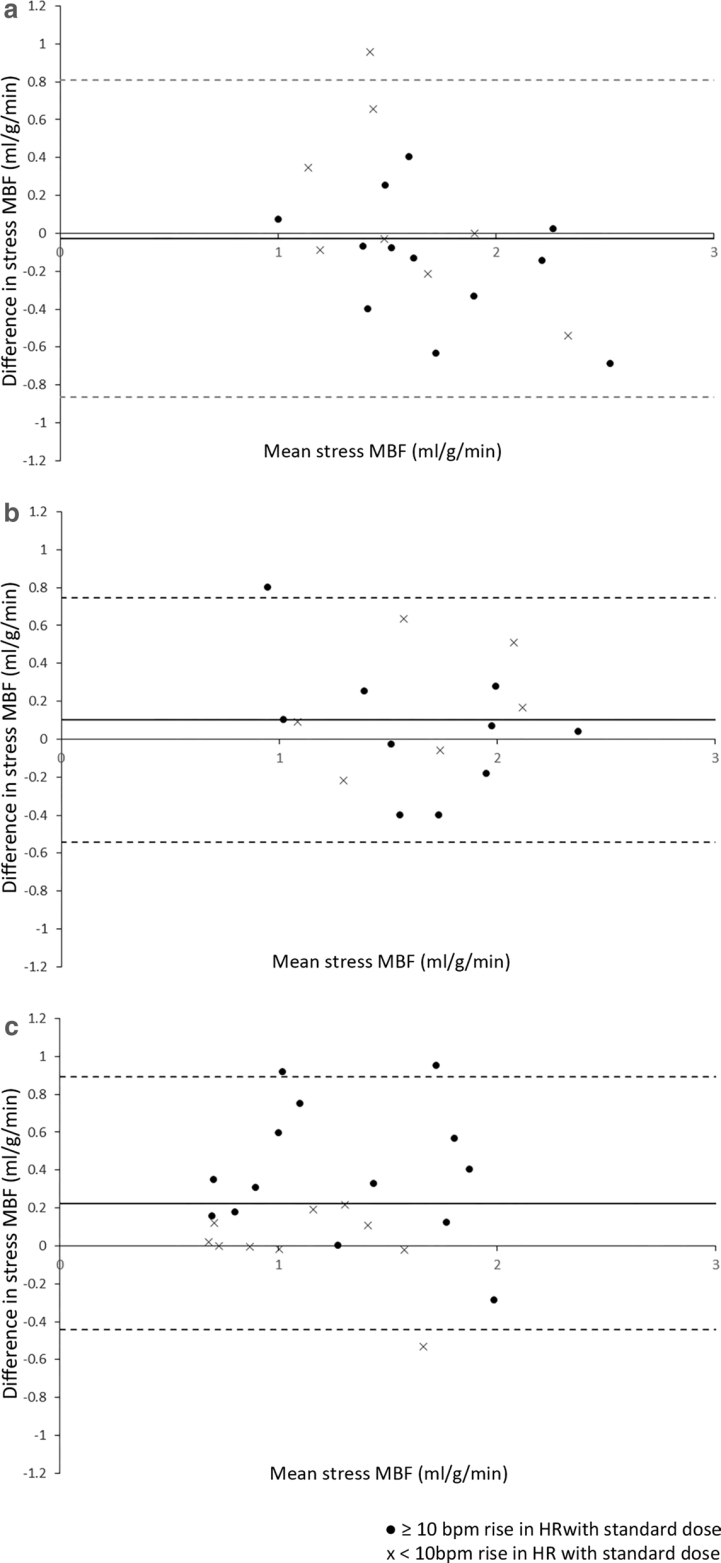
Difference in MBF between doses. ● represents adequate HR response to standard dose adenosine, x represents blunted response. In Group 3, stress MBF is significantly higher following high dose adenosine, this effect does not appear to be related to HR response at standard dose

Bland Altman plots (Fig. 4) show the spread of differences in MBF between adenosine doses and those with and without adequate response to adenosine at standard dose.

There were weak correlations between increase in HR and MPR both with standard (r = 0.266, p = 0.045) and high dose adenosine (r = 0.54, p < 0.001). HR response with standard dose adenosine did not correlate with an increase in MBF or MPR with high dose adenosine.

Five patients demonstrated failed splenic switch off with standard dose adenosine (Table 3). Analysis was repeated, excluding those with failed splenic switch off. These showed the same pattern with no difference in stress MBF or MPR between standard and high dose adenosine in Group 1 and Group 2, but higher stress MBF (1.15 ± 0.46 vs 1.38 ± 0.45, p = 0.009) and MPR (1.99 ± 0.88 vs 2.34 ± 0.91, p = 0.009) in Group 3.

### Subgroup analyses

#### Heart rate response to adenosine

Patients were divided into groups of non-responders (n = 24) and responders (n = 36) based on a HR increase of < 10 bpm or ≥ 10 bpm. There was no significant difference in LVEF, age or incidence of diabetes or beta blocker usage between the two groups. Stress HR was significantly different between groups both with standard and high dose adenosine (70 ± 14 vs 88 ± 15 bpm, p =  < 0.001 at standard dose and 75 ± 12 vs 89 ± 18 bpm, p = 0.001 at high dose). There was no significant difference in rest HR or stress MBF between the two groups at either adenosine dose.

In the non-responder group, stress HR was significantly higher with high dose adenosine than with standard dose (Table 4). Those with adequate HR response to standard dose did not have a significant increase in stress HR between doses. No significant difference was seen in stress MBF between standard and high dose adenosine regardless of adequate HR response to standard dose adenosine.

**Table 4:** Difference in response to adenosine doses divided by HR response to standard dose

	Standard dose	High dose	p
Non-responders (n = 24)			
Rest HR (bpm)	67 ± 16	68 ± 14	0.789
Stress HR (bpm)	70 ± 14	75 ± 12	0.034
Increase in HR (bpm)	3 ± 6	7 ± 11	0.053
Stress MBF (ml/g/min)	1.35 ± 0.49	1.45 ± 0.46	0.188
MPR	1.93 ± 0.77	2.05 ± 0.70	0.215
Adequate HR response (n = 36)			
Rest HR (bpm)	69 ± 12	70 ± 13	0.461
Stress HR (bpm)	88 ± 15	89 ± 18	0.762
Increase in HR (bpm)	19 ± 9	19 ± 13	0.838
Stress MBF (ml/g/min)	1.48 ± 0.60	1.59 ± 0.43	0.103
MPR	2.32 ± 0.86	2.53 ± 0.63	0.073

Stress HR was significantly higher with high dose adenosine in the non-responder group only. Non-responders and responders were defined by heart rate increase with adenosine of < 10 bpm or ≥ 10 bpm. There data are from patients only. No difference was seen in stress MBF in either group

Data given as mean ± standard deviation or n (%)

*HR* heart rate, *MBF* myocardial blood flow

#### Ischaemia

Within group 1, 11 patients had evidence of regional inducible ischaemia. On visual analysis, ischaemia was seen in the same coronary territories between the adenosine doses. A total of 60 ischaemic segments were visually identified following standard dose adenosine, and 63 segments following high dose. No significant difference was seen in MBF in between standard and high dose adenosine in either the ischaemic (1.46 ± 0.30 vs 1.48 ± 0.40 ml/g/min, p = 0.697) or non-ischaemic segments (1.72 ± 0.57 vs 1.77 ± 0.52 ml/g/min, p = 0.130) in these patients.

## Discussion

The Society for Cardiovascular Magnetic Resonance Standardized (SCMR)  imaging protocols for stress perfusion recommend an adenosine dose of 140 μg/kg body weight/min for 2–4 min with an increase in the dose if there is inadequate HR and SBP response [5]. Our results inform several aspects of this recommendation: the duration of adenosine infusion, the dose of adenosine and the use of HR and SBP as indicators of adequate response. In healthy controls, increased dose or extended duration adenosine were not associated with significant changes in stress MBF compared with standard dose adenosine. Equally, in patients with normal or mildly impaired LV systolic function there was no effect of higher dose adenosine, but in those with moderate to severe LV systolic dysfunction (LVEF ≤ 40%), higher dose adenosine produced higher stress MBF. We further show that HR and SBP are unreliable markers of haemodynamic response.

### Duration of adenosine infusion

The duration of adenosine infusion has not previously been studied for stress perfusion CMR but has been the subject of studies in nuclear cardiology. In Single Photon Emission Computed Tomography, a 3-min adenosine infusion showed better tolerability with similar diagnostic performance compared with a 6-min protocol[17]. A PET study using Rb-82 compared several adenosine regimes in 127 subjects and found that a 6-min adenosine infusion protocol with Rb-82 activation at 3 min was associated with 11.4% higher stress MBF and 15.7% higher coronary flow reserve (CFR) than a 4-min adenosine infusion with Rb-82 activation at 2 min [18]. Further extension of the adenosine infusion time prior to Rb-82 activation did not increase increased stress MBF or CFR further. These results are not directly applicable to CMR due to the differences in tracer kinetics and data capture between PET and CMR. Our data show that in myocardial perfusion CMR, there is no significant difference in haemodynamic response and no change in quantitative MBF between a 4 min and an extended 8 min adenosine protocol in healthy subjects. These results suggest that the shorter duration protocols that are in current clinical use and recommended in current guidance are adequate for myocardial perfusion CMR.

### Dose of adenosine infusion

The dose of adenosine infusion has been studied more extensively, using multiple modalities. Early invasive studies using intracoronary Doppler assessment of coronary blood flow velocity and total coronary resistance showed that intravenous adenosine at doses of 140 µg/kg/min resulted in maximal hyperaemia, defined by papaverine response, in 84% of subjects [3]. Several invasive studies have assessed the effect of adenosine dose on FFR with higher doses showing no significant change in FFR compared with lower doses [19–21]. In a CMR study, Karamitsos et al. showed that a stepwise increase in the adenosine dose from 140 μg/kg/min to 210 μg/kg/min is safe and increases the rate of patients with an adequate haemodynamic response [22]. However, the MBF response to different adenosine doses has not previously compared using quantitative myocardial perfusion CMR. In addition to studying the same individuals repeatedly, we obtained MBF values at different adenosine doses in the same imaging session. This approach overcomes the potential confounders of day-to-day physiological variation in haemodynamic response and allows direct comparison of dose effects. Our data show no significant difference in MBF following standard and high dose adenosine in healthy subjects and patients with CAD or heart failure with LVEF ≥ 40%, suggesting that irrespective of haemodynamic response, standard dose adenosine in these groups reliably induces maximal hyperaemia.

### Impaired LV systolic function

In patients with severe systolic impairment, previous studies have shown a blunted HR response to adenosine, with an increase in adenosine from 140 to 210 µg/kg/min more commonly required to achieve a sufficient haemodynamic response [8]. In CMR, a previous study reported LVEF < 57% as an independent predictor of inadequate haemodynamic response to standard adenosine dose [22]. Within our patient cohort, there was no correlation between LVEF and HR rise, and no difference in HR rise between the patient groups, or any significant difference in the rate of non-responders. However, our study showed for the first time that among patients with heart failure and significant LV systolic impairment (LVEF ≤ 40%), stress MBF increases with higher doses of adenosine, suggesting that standard dose regimes fail to induce maximal hyperaemia and are not appropriate in these patients. A pattern of decreased response to adenosine in heart failure requiring higher doses to achieve stress has previously been suggested [8]. Potential mechanisms for the lower adenosine effect include the downregulation of gene expression of both adenosine receptors and adenosine deaminase in impaired myocardium, together with a decrease in the activity of adenosine deaminase [23, 24]. Increased levels of cardiac adenosine have also been measured in chronic heart failure patients, and this higher endogenous level may explain the requirement for higher exogenous doses to achieve the anticipated vasodilation required in stress testing [23, 24].

### Haemodynamic response

In non-invasive testing, response to adenosine and the achievement of hyperaemia is commonly assessed using haemodynamic response relating to peripheral vasodilation. Conventionally, an increase in HR by > 10 bpm and a fall in systolic SBP by > 10 mmHg are considered markers of adequate hyperaemia [5]. A small previous PET perfusion study suggested a correlation between HR response and stress MBF [18], but confounders such as LVEF were not explicitly considered. In an earlier larger PET study, change in HR correlated poorly with stress MBF, and not with CFR, leading the authors to suggest that peripheral haemodynamic changes could not be used to assess the adequacy of response to adenosine [6]. No CMR studies have previously looked at haemodynamic response and change in quantitated MBF.

Within our study, 61% of those patients with LVEF > 40% reached the threshold of 10 bpm, comparable to published results in other studies with a similar patient group [8]. This relatively low response rate may be due to the presence of medications and other co-morbidities in our patient cohort. Blunted haemodynamic response to adenosine has been reported in diabetes, beta-blocker usage and CAD as well as impaired LVEF [8–10, 22, 25]. Increase in HR correlated only weakly with MPR and no significant difference was seen in stress MBF between groups classified by HR response.

This study showed no significant relationship between rise in HR at standard dose adenosine and an increase in stress MBF or MPR with high dose adenosine. This indicates that in patients with a low HR response at standard dose adenosine, a higher dose does not increase myocardial perfusion—a finding that questions the validity of current guideline recommendations and widely used clinical practice.

Further, we saw no significant difference in SBP change between groups of patients, or adenosine doses and < 3% of patients had a decrease in SBP of ≥ 10 mmHg as described in standard protocols. SBP even increased in healthy subjects over baseline. These data suggest, in keeping with previous studies, that in particular SBP response should not be used as a marker of adequate vasodilator response, possibly due to an adrenergic response to adenosine symptoms, which overcomes the vasodilator effects on blood pressure.

### Clinical implications

Our data suggest that those with reduced LVEF should have higher dose adenosine to achieve maximal hyperaemia, regardless of haemodynamic response. Our data also question the use of HR and SBP response to standard dose adenosine as criteria to increase the adenosine dose as it does not appear to increase MBF. However, due to low numbers of patients with inducible ischaemia in this study we have not been able to assess the diagnostic impact of our observations although we observed a small, non-significant, increase in the number of ischaemic segments identified following high dose adenosine.

### Limitations

Our data may be influenced by physiological variation, although we have tried to minimise this. It is possible some effects may not have been controlled for, although we have previously demonstrated no significant difference in serial measurements of stress MBF within a CMR study [12]. Caffeine has been demonstrated to affect adenosine stress perfusion CMR [26]. Although we advised our subjects to avoid caffeine for 24 h prior to the scan, previous studies have demonstrated that up to 20% may still have detectable caffeine levels [27] and we cannot account for how these may be distributed between our patient groups in this study. The age range of our healthy subjects was considerably lower than those of the patient groups, if age influences the response to adenosine then the results from these healthy subjects may not be applied to our patient groups. There was no significant difference in mean ages between the patient groups. Our data cannot exclude that a higher dose of adenosine than 210 µg/kg/min might further increase stress MBF in those with LVEF < 40%, but our data also cannot be extrapolated to support this possibility. Although the use of doses in excess of 210 mcg/kg/min are not used in routine practice, future studies should explore higher doses in particular in HF patients.

## Conclusions

Increasing adenosine dose is well tolerated and related to increased stress myocardial blood flow in patients with significant LV impairment. Achievement of adequate myocardial vasodilator response, assessed by quantitative perfusion, is not significantly related to peripheral haemodynamic measurements SBP and HR. These observations may impact future practice guidelines for stress perfusion CMR. Dosage of adenosine in clinical perfusion assessment should be carefully considered and may need to be increased in subsets of patients, in particular those with severely impaired LV function, or alternative stress agents considered.

## Data Availability

The datasets used and/or analysed during the current study are available from the corresponding author on reasonable request.

## Abbreviations

BMI: Body mass index
CAD: Coronary artery disease
CFR: Coronary flow reserve
CMR: Cardiovascular magnetic resonance
FFR: Fractional flow reserve
HF: Heart failure
HR: Heart rate
LGE: Late gadolinium enhancement
LV: Left ventricle/left ventricular
LVEF: Left ventricular ejection fraction
MBF: Myocardial blood flow
MPR: Myocardial perfusion reserve
PET: Positron emission tomography
SBP: Systolic blood pressure
